# Asymptomatic secondary hyperparathyroidism can mimic sacroiliitis on computed tomography

**DOI:** 10.1038/s41598-021-83989-1

**Published:** 2021-02-22

**Authors:** Virginie Kreutzinger, Torsten Diekhoff, Lutz Liefeldt, Denis Poddubnyy, Kay Geert A. Hermann, Katharina Ziegeler

**Affiliations:** 1 Department of Radiology, Vivantes Klinikum im Friedrichshain, Berlin, Germany; 2 Department of Radiology, Charité-Universitätsmedizin Berlin, Berlin, Germany; 3 Department of Nephrology and Internal Intensive Care Medicine, Charité-Universitätsmedizin Berlin, Berlin, Germany; 4 Department of Rheumatology, Charité-Universitätsmedizin Berlin, Berlin, Germany

**Keywords:** Musculoskeletal system, Skeleton, Skeleton, Phosphorus metabolism disorders

## Abstract

Secondary hyperparathyroidism (sHPT) as a result of chronic kidney disease (CKD) is a common health problem and has been reported to manifest at the sacroiliac joints (SIJ). The aim of this investigation was to systematically assess sacroiliac joint changes in asymptomatic sHPT as detected by high-resolution CT. Included in this IRB-approved retrospective case–control study were 56 patients with asymptomatic sHPT as well as 259 matched controls without SIJ disease. Demographic data were retrieved from electronic patient records. High-resolution computed tomography datasets of all patients were subjected to a structured scoring, including erosions, sclerosis, osteophytes, joint space alterations and intraarticular calcifications. Chi^2^ tests were used to compare frequencies of lesions. Erosions were significantly more prevalent in patients with sHPT, and were found mainly in the ventral (28.6% vs. 13.9%; p = 0.016) and middle (17.9% vs. 7.7%; p = 0.040) iliac portions of the SIJ. Partial ankylosis was rare in both cohorts (3.6% vs. 5.0%; p > 0.999); complete ankylosis was not observed. Neither extent not prevalence of sclerosis or calcifications differed significantly between groups. Joint lesions reminiscent of sacroiliitis can be found in a substantial portion of asymptomatic patients with secondary hyperparathyroidism. Further investigations into the clinical significance of these findings are warranted.

## Introduction

Chronic kidney disease (CKD) is a global health problem, estimated to affect up to 10% of the general population, with a rising prevalence over the last decades^1,2^. A frequent complication in patients requiring haemodialysis is the development of osteo-articular disease^3^, especially secondary hyperparathyroidism (sHPT)^4^. Parathyroid hormone (PTH) has a catabolic effect on bone metabolism and has been shown to decrease bone-mineral density of cortical bone^5^; longstanding elevated PTH levels may lead to osteitis fibrosis cystica, a high turnover bone disease presenting with lytic lesions on imaging as a result of replacement of mineralized bone with fibrous tissue^6^. These findings are most likely the reason why HPT is sometimes considered a differential diagnosis in sacroiliitis imaging^7^, as subchondral bone resorption may be difficult to distinguish from true erosions on imaging. Additionally, HPT is an important predisposing factor of calcium pyrophosphate dihydrate deposition (CPPD)^8^, which in turn manifests at the sacroiliac joints in up to 50% of CPPD patients and may cause bilateral erosions, joint space narrowing and sclerosis^9,10^.

A recent study by Tezcan et al. has investigated MRI features of asymptomatic primary hyperparathyroidism in 49 patients^11^ and found bone marrow edema in 16.3% of patients, though no significant difference to healthy controls could be detected. In terms of secondary hyperparathyroidism, published evidence of manifestations at the SIJs to date is limited to case reports^12^, investigations undertaken more than 30 years ago^13^ or radiographic studies^14^.

To our knowledge, no systematic investigation on the pattern of arthropathy of the SIJ in sHPT as detected by computed tomography has been undertaken thus far. The aim of this study was to systematically describe the pattern of arthropathy in asymptomatic secondary hyperparathyroidism compared the normal population.

## Materials and methods

### Ethical approval and patient consent

Prior to data acquisition, approval was attained from the ethics review board of the Charité Universitätsmedizin Berlin (EA1/300/19). Due to the retrospective nature of the investigation the ethics review board waived individual written informed consent. Consent for use of de-identified imaging data in scientific publications was obtained as a routine practice in our institution. The study was conducted in compliance with the Declaration of Helsinki and local legislation and ethical standards.

### Patients

Included into the case-group of this retrospective case–control study were patients with known secondary hyperparathyroidism who had received a computed tomography of abdomen and pelvis between March 2016 and March 2019. Clinical data was retrieved from electronic patient records. Patients with known disease of the sacroiliac joints, known rheumatic disease, cutaneous psoriasis, inflammatory bowel disease, uveitis, malignancy of the skeletal system (both primary and metastatic) and fractures of the pelvis were excluded. The patients were matched with subjects from an existing, retrospectively acquired cohort from our institution, who had undergone imaging during the same period and for whom the same clinical information (except parameters of sHPT) were available. Indications for CT examinations in the case group were oncological staging (15/56), infection (10/56), trauma/bleeding (5/56) and other, including evaluation before kidney donation as recipient (27/56). Indications for CT examination in the control group were oncological staging (130/259), infection (83/259), trauma/bleeding (6/259) and other, including evaluation before kidney donation (27/56). Matching was performed in a ration of 4 controls for every case.

### Imaging technique

For all CT scans a special reconstruction of the pelvic skeleton was available, yielding images in a similar quality as in dedicated SIJ imaging. All image datasets were scored by one radiological resident with 7 years of experience in MSK imaging (KZ), blinded for all clinical data. Images were read using dedicated software (Horos v3.3.6, The Horos Project, public license) in random order and predominantly in oblique-coronal and axial orientation, using bone-window settings.

### Scoring system

Expanding on previous work of our research group^15^, a scoring system was used that divides the sacroiliac joints into 12 joint regions on each side. In each region, erosions and sclerosis were assessed in a categorical fashion, laid out in Table 1.

**Table 1:** Scoring system. Overview of scoring system.

	Erosions	Sclerosis	Joint space	Osteophytes	Calcifications
0	None	None	Normal	None	None
1	1–2 erosion	Possible/very little sclerosis	Possible widening/narrowing	Small osteophyte (≤ 5 mm)	Few or punctuate calcifications
2	3–5 erosions	Marked sclerosis	Pseudo-widening/-narrowing	Larger osteophyte (> 5 mm)	Extensive or linear calcifications
3	> 5 erosions		Partial ankylosis	Bridging osteophyte	
4			Complete ankylosis		

Additionally, joint space alterations (including pseudo-widening and ankylosis) and intraarticular calcifications (both per side) as well as osteophytes (ventral and dorsal, separately for each side) were scored. Prior to scoring, an atlas was assembled from example patients not included in this analysis, which was used as a reference during scoring. A teaching session on 15 test cases (not included in the study) was carried out with a consultant radiologist with expertise in MSK radiology (TD) before commencement of the scoring process. In order to calculate inter- and intra-reader reliability, a random sample of 50 study patients was scored by both junior (KZ) and senior reader (TD)—in case of the junior reader a second time.

### Statistical analysis

Statistical analyses were carried out using SPSS Version 25 (IBM Corporation, New York, USA). Patients were matched with controls using the dedicated propensity score matching tool; age, gender and weight were chosen as covariables, with a defined tolerance of 0.01. Scoring results were summarised as sum scores for each structural lesion separately; on the patient level, positivity for erosions was defined as a sum score ≥ 2. Frequencies of structural lesions were compared using Chi^2^ tests. Sum scores of structural lesions were compared using Mann–Whitney-U tests. Intraclass-correlation coefficients were calculated for inter- and intra-reader reliability between readers, using a two-way mixed model ICC(3,2)^16^ on sum scores for each lesion type. A significance level of p < 0.05 was assumed for all tests.

## Results

### Patients’ characteristics

**Figure 1:**
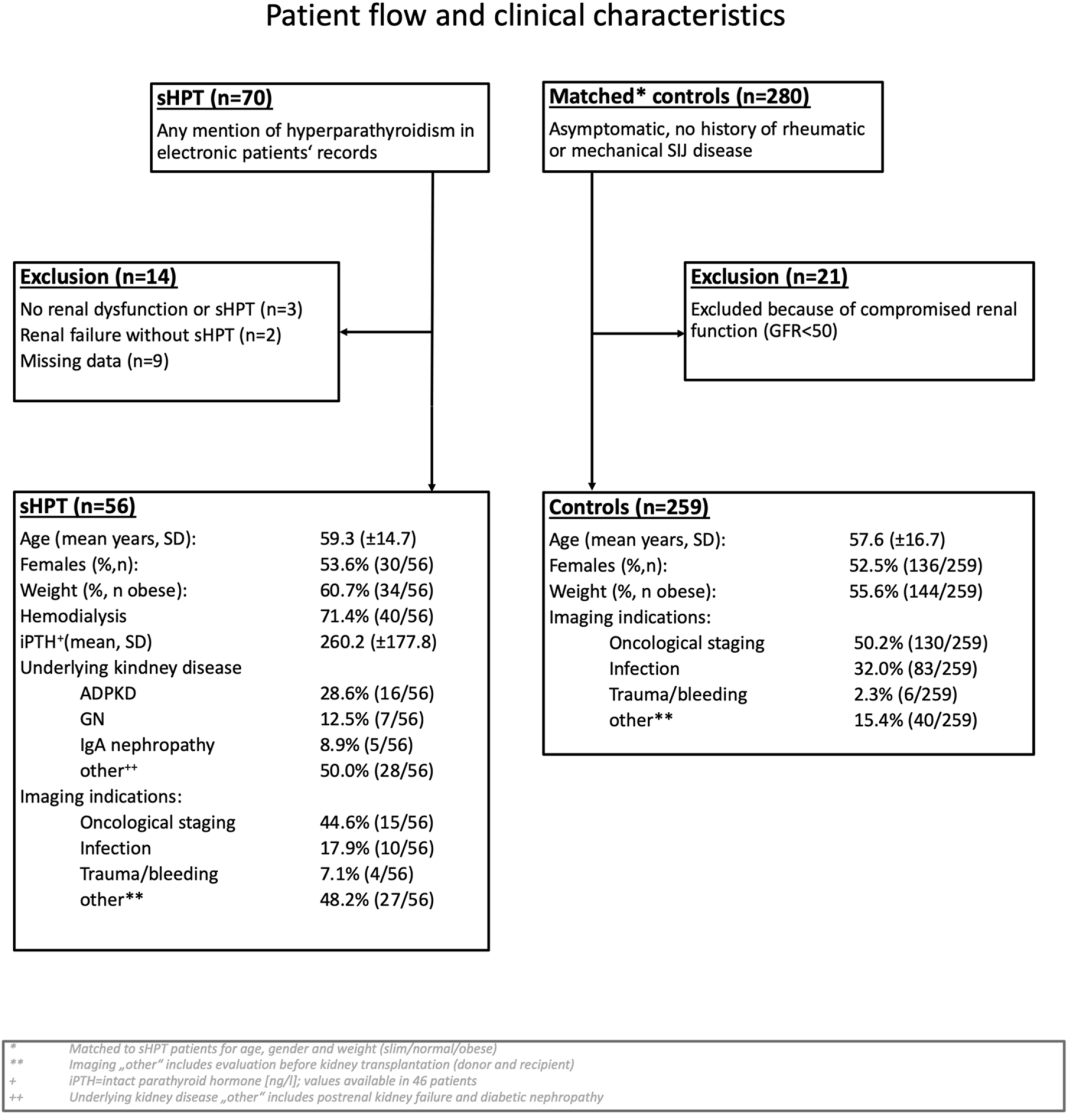
Patient flow. *sHPT *secondary hyperparathyroidism, *GFR *glomerular filtration rate, *SD *standard deviation, *iPTH *intact parathyroid hormone, *ADPKD *autosomal dominant polycystic kidney disease, *GN *glomerulonephritis. Distribution of clinical characteristics was compared between sHPT and controls with appropriate statistical tests (t-test, Chi^2^ test)—no significant differences were detected.

A total of 315 patients (56 sHPT, 259 controls) were included in this investigation; a summary of patient flow and clinical characteristics is provided as Fig. 1. As per study design, mean age and gender distribution did not differ between groups.

### Frequency and distribution of structural lesions

A complete table of frequencies of structural lesions as well as mean sum scores is provided as Table 2.

**Table 2:** Structural CT lesions in secondary hyperparathyroidism compared with controls. *sHPT *secondary hyperparathyroidism, *SD *standard deviation. Significantly (p < 0.05) values are printed in bold. P-values were derived from Chi^2^ tests for nominal and Mann–Whitney-U tests for continuous data.

Lesion type		sHPT (n = 56)	Controls (n = 259)	p-values
Erosion	Erosion score (0–72), mean (SD)	**2.6 (7.4)**	0.6 (2.7)	0.001
	Erosion ventral ilium present, n (%)	16 (28.6)*	36 (13.9)	0.016
	Erosion ventral sacrum present, n (%)	6 (10.7)	12 (4.6)	0.105
	Erosion middle ilium present, n (%)	10 (17.9)*	20 (7.7)	0.040
	Erosion middle sacrum present, n (%)	2 (3.6)	2 (0.8)	0.147
	Erosion dorsal ilium present, n (%)	6 (10.7)*	9 (3.5)	0.033
	Erosion dorsal sacrum present, n (%)	3 (5.4)*	1 (0.4)	0.019
Sclerosis	Sclerosis score (0–48), mean (SD)	3.0 (3.0)	3.5 (3.2)	0.300
	Sclerosis ventral ilium present, n (%)	34 (60.7)	151 (58.3)	0.767
	Sclerosis ventral sacrum present, n (%)	9 (16.1)	68 (26.3)	0.124
	Sclerosis middle ilium present, n (%)	15 (26.8)	74 (28.6)	0.871
	Sclerosis middle sacrum present, n (%)	2 (3.6)	14 (5.4)	0.746
	Sclerosis dorsal ilium present, n (%)	14 (25.0)	91 (35.1)	0.162
	Sclerosis dorsal sacrum present, n (%)	8 (14.3)	45 (17.4)	0.695
Joint space alterations	Joint space alterations score, mean (SD)	0.6 (1.3)	0.4 (1.3)	0.586
	Pseudo-widening present, n (%)	3 (5.4)	4 (1.5)	0.110
	Partial ankylosis present, n (%)	2 (3.6)	13 (5.0)	> 0.999
Osteophytes	Osteophyte score (0–12), mean (SD)	1.5 (2.0)	2.3 (2.3)*	0.020
	Osteophyte ventral present, n (%)	18 (32.1)	102 (39.4)	0.364
	Osteophyte dorsal present, n (%)	18 (32.1)	134 (51.7)*	0.008
Calcification	Calcification score, mean (SD)	0.6 (1.1)	0.4 (0.9)	0.091
	Calcification present, n (%)	15 (26.8)	45 (17.4)	0.132

**Figure 2:**
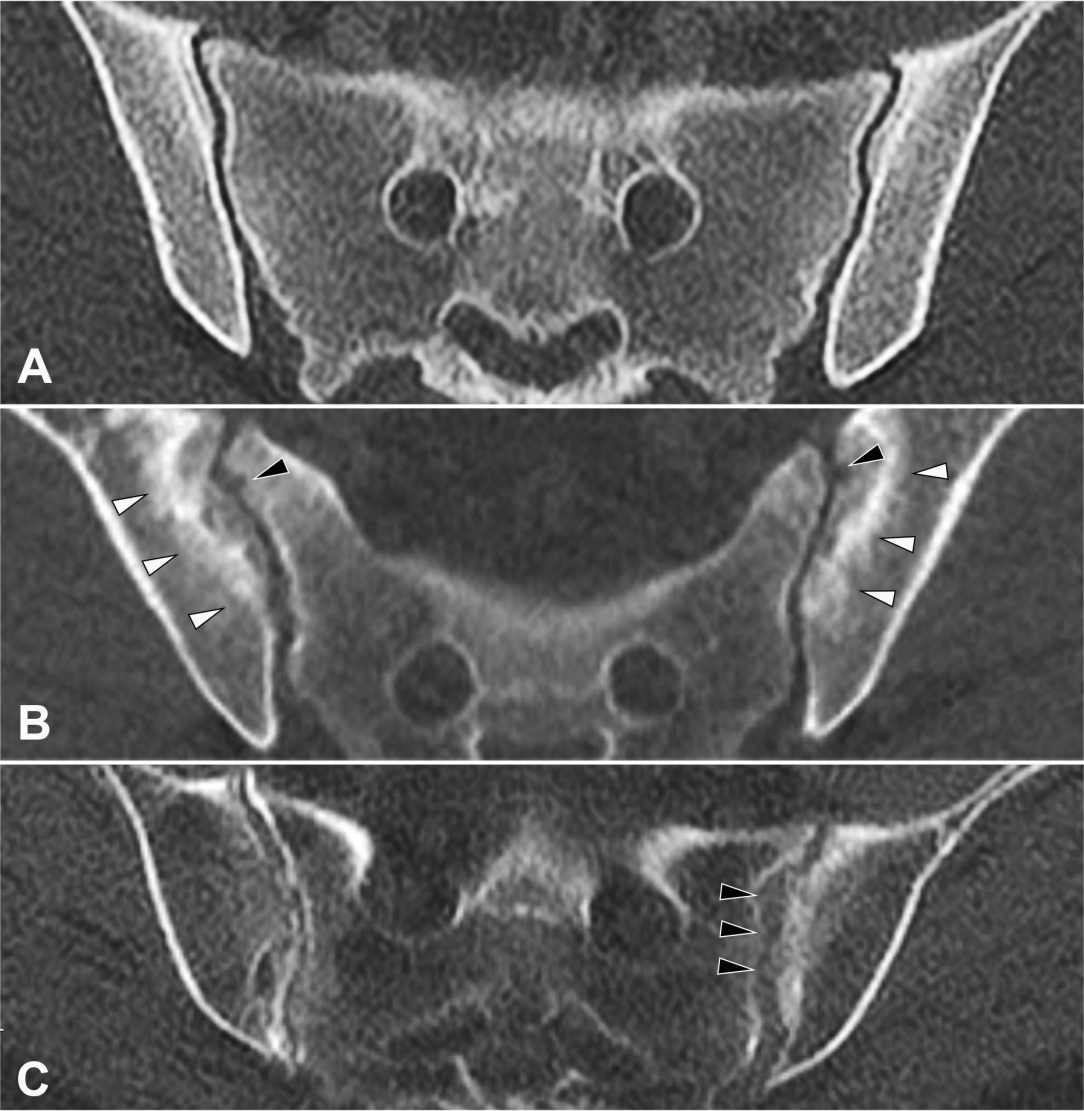
Example of joint alterations in sHPT. Axially reconstructed high-resolution CT images. (**A**) Healthy control patient with normal SIJ. (**B**) Patient with secondary hyperparathyroidism: note the subchondral bone resorption mimicking erosions (black arrowheads) as well as the sclerotic rim around the resorption zone (white arrowheads). (**B**) Patient with secondary hyperparathyroidism: note the irregular, pseudo-widened left joint space (black arrowheads) compared to the regular joint space on the right.

Erosions were observed in significantly more sHPT patients than their matched controls (11/56 vs. 10/259; p < 0.001). The difference was most pronounced in the iliac joint portions with 28.6% vs. 13.9% ventrally (p = 0.001), 17.9% vs. 7.7% in the middle (p = 0.040) and 10.7% vs. 3.5% dorsally (p = 0.033). Neither sclerosis nor joint space alterations including partial ankylosis differed in frequency and extent between sHPT patients controls; there was no instance of complete ankylosis in either cohort of this investigation. Intraarticular calcifications were seen in 26.8% of sHPT patients and 17.4% of controls—the difference was not statistically significant (p = 0.132). Osteophytes were more prevalent in controls than sHPT patients with 51.7% vs. 32.1% (p = 0.008) in the dorsal aspect of the joint. Imaging examples from the patient cohort are supplied as Fig. 2.

### Inter-and intra-reader agreement

Interreader reliability, interpreted according to Koo et al.^16^, was moderate for erosions (0.57; 95% CI 0.25–0.76; p = 0.002), good for sclerosis (0.78; 95% CI 0.64–0.87; p < 0.001), moderate for joint space alterations (0.74; 95% CI 0.53–0.85; p < 0.001), good for osteophytes (0.83; 95% CI 0.71–0.91; p < 0.001) and good for calcifications (0.85; 95% CI 0.74–0.91; p < 0.001). Intrareader reliability was good for erosions (0.77; 95% CI 0.59–0.87; p < 0.001), good for sclerosis (0.88; 95% CI 0.78–0.93; p < 0.001), good for joint space alterations (0.82; 95% CI 0.68–0.90; p < 0.001), good for osteophytes (0.87; 95% CI 0.77–0.93; p < 0.001) and good for calcifications (0.86; 95% CI 0.76–0.92; p < 0.001).

## Discussion

To our knowledge the data presented here is the first systematic exploration of structural lesions of the sacroiliac joints of patients with asymptomatic sHPT. We found a significantly higher prevalence of erosions in sHPT patients, while ankylosis and sclerosis were equally rare in both groups. These findings carry significance in the context of sacroiliitis imaging, where erosions are considered a specific finding of inflammatory joint disease.

Our findings somewhat contradict those of Tezcan et al.^11^, who examined inflammatory MRI lesions in primary hyperparathyroidism and did not find significant differences between HPT patients and controls. The most likely explanation for this incongruence is that standard SIJ-MRI imaging has known deficits in the depiction of small structural lesions^17^, such as erosions. Osteophytes, which are considered degenerative rather than inflammatory joint lesions were significantly less prevalent in the sHPT group. This is surprising, as osteoarthritis is considered a common finding in patients with advanced CKD^3^, and patients were matched with regards to predisposing factors for mechanical SIJ disease such as obesity and parity in women^18^. A possible explanation may be a comparably larger proportion of immobile patients in the sHPT group. Another unexpected finding is the similar prevalence of joint space calcifications in both groups—as hyperparathyroidism is a known risk factor for developing CPPD^8^, a comparably higher percentage of sHPT patients with articular calcifications would have been expected. In our opinion, these findings support the hypothesis, that SIJ changes in sHPT are most likely manifestations of osteitis fibrosis cystica rather than sacroiliac CPPD.

Due to the retrospective nature of the investigation, data on back pain in sHPT patients could only be captured from electronic patients records, so that we cannot exclude the possibility that some of the patients may in fact have symptomatic joint disease, somewhat limiting the validity of our results. The results are limited further by the small size of the patient group—larger, prospective cohorts with more detailed information on severity and duration of the secondary hyperparathyroidism, including information on vitamin D, calcium and alkaline phosphatase are needed to illicit the factors that contribute to the development of the lesions described in this analysis.

In conclusion, secondary hyperparathyroidism can mimic sacroiliitis on SIJ CT in a substantial portion of patients and should be considered when assessing joints for inflammatory changes. Further investigations into the clinical significance of these lesions for affected patients are warranted.

## Data Availability

The datasets generated during and/or analysed during the current study are available from the corresponding author on reasonable request.
